# The cornerstones of *Cerebellum and Ataxias*: from peer review to rapid visibility in a rising discipline

**DOI:** 10.1186/2053-8871-1-1

**Published:** 2014-06-16

**Authors:** Mario Manto

**Affiliations:** Unité d’Etude du Mouvement (UEM), FNRS ULB-Erasme, 808 Route de Lennik, 1070 Bruxelles, Belgium

**Keywords:** Cerebellum, Ataxias, Peer-review, Open access, Visibility, Journal

Several questions arise immediately when a new scientific journal emerges from the ground: why a new journal? What makes this journal special? Will the peer-review process be handled in a rigorous and fair manner? Will the journal be visible and accessible to all? How fast will the publication process be? What will be the cost of publishing in the journal?

## Our scope in a rapidly changing discipline, which is now recognized

The neuroscientists and clinicians working in the field of cerebellar research and ataxiology are living in a fascinating period: the recognition by the scientific community of the specificity of their activity. Neglected for many years, the field of cerebellar ataxias is now evolving as a well-circumscribed neuroscience and clinical discipline. Disorders of the cerebellum present with specific symptoms and signs, have a specific natural course and particular mechanisms in terms of pathogenesis. It can be predicted that most university centers in the world will host an ataxia unit in a decade from now. Indeed, numerous clinical disorders affect the cerebellum at any stage of life, either in an acute or in a chronic way. Cerebellar operations spread from motor to cognitive and behavioral aspects [1, 2]. With its unique geometrical structure, the region of the brain containing the most numerous neurons is more than ever a topic of interest. Indeed, cerebellum maps to cerebral association networks in a highly organized manner, mirroring the asymmetries of the cerebral cortex for language and attention [3]. The “little brain” is scrutinized with the hope that it will reveal its numerous secrets, and that these secrets will have numerous applications. Thanks to next generation sequencing techniques, the discoveries in genetics are enriching the basket of cerebellar disorders nearly on a weekly basis. A convergence of molecular discoveries and clinical observations is appearing [4]. However, numerous case descriptions and case series remain unpublished, despite their importance for daily clinical care, or their relevance for building clinically-relevant models and the successful implementation of personalized molecular medicine tailored to individuals [5]. We aim to attract these reports, for the benefit of all the community. And the patients themselves.

*Cerebellum and Ataxias* will consider not only scientific reports, but also clinical cases of the numerous forms of ataxias affecting children and adults. Case reports and clinical series, as well as reviews, commentaries and teaching images with a take-home message will be welcome.

## The major features of *Cerebellum and Ataxias*: high quality peer-review, very fast turnaround times, high visibility and immediate online availability

*Cerebellum and Ataxias* aims to publish very high quality articles in the field of cerebellar research and ataxias. The international group of experienced and renowned editors will ensure the rigor of the peer-review process. They have a strong personal experience in cerebellar research and ataxias. All of them are referees for the highest-ranking scientific journals in the world, and are thus very familiar with the peer-review process. They all know that critical and independent evaluation of the manuscripts is essential to building a journal that (a) will impact quickly on scientific literature, and (b) will be useful and meaningful to scientists and clinicians. Importantly, *Cerebellum and Ataxias* aims to interact closely and very fairly with authors, acting with efficiency and transparency [6]. Many PhD students - who will become the laboratory or clinical leaders in two or three decades - complain when their submission to a journal is in a ‘deadlock’ or delayed. Our publication process will be very fast, with a maximum of three weeks from the submission to the first decision, and a very short delay from the acceptance to the publication. Moreover, the journal will have great online visibility. Thanks to the open access model, all the articles will be available immediately and universally to a very large readership. As such, *Cerebellum and Ataxias* will be invaluable to students, scientists and clinicians having an interest in the cerebellum and diseases affecting its functions. We aim to build a journal where readers will find easily both practical and theoretical information impacting on practice, including therapies.

The open access model has a great advantage: the copyright of published articles belongs to authors. This model is associated with an article-processing charge (APC). Several funding agencies cover the APC. In addition, the list of institutions with BioMed Central membership is continuously growing (see http://www.biomedcentral.com/inst/). Authors are invited to check whether their institution is member, since the APC might be fully or partly covered. It is one of our goals to try to maintain the APC as affordable as possible.

We are extremely lucky to work with a top team at BioMed Central. We express our warmest thanks to the editorial staff, especially Nandita Quaderi and Elizabeth Bal, for their expertise, their high degree of professionalism and their continuous support. *Cerebellum and Ataxias* will work very closely with its cousin journal *The Cerebellum*, whose editor Martijn Roelandse plays an instrumental role in its development. The two journals will be complementary. As such, in addition to direct submissions to *Cerebellum & Ataxias*, manuscripts originally reviewed by *The Cerebellum* can be transferred directly, should the authors request it, to *Cerebellum & Ataxias.* This will avoid delays in publication and the cumbersome task of resubmitting the same materials.

*Cerebellum and Ataxias* will face a challenge: to rapidly establish itself as an online platform allowing readers to get immediate access to the fast pace of changes in the field of cerebellar disorders. We aim to succeed in this challenge of building *an ultrafast and meaningful source of information* for the cerebellum community, and even more. On behalf of the Editorial Board, we now look forward to your submissions and feedback!
